# Integrated Transcriptome and Metabolome Analysis to Identify Sugarcane Gene Defense against Fall Armyworm (*Spodoptera frugiperda)* Herbivory

**DOI:** 10.3390/ijms232213712

**Published:** 2022-11-08

**Authors:** Ao-Mei Li, Miao Wang, Zhong-Liang Chen, Cui-Xian Qin, Fen Liao, Zhen Wu, Wei-Zhong He, Prakash Lakshmanan, You-Qiang Pan, Dong-Liang Huang

**Affiliations:** 1 Key Laboratory of Sugarcane Biotechnology and Genetic Improvement (Guangxi), Ministry of Agriculture and Rural Affairs, Guangxi Key Laboratory of Sugarcane Genetic Improvement, Sugarcane Research Institute, Guangxi Academy of Agricultural Sciences, Nanning 530007, China; 2 Shenzhen Branch, Guangdong Laboratory of Lingnan Modern Agriculture, Genome Analysis Laboratory of the Ministry of Agriculture and Rural Affairs, Agricultural Genomics Institute at Shenzhen, Chinese Academy of Agricultural Sciences, Shenzhen 518000, China

**Keywords:** sugarcane, *Spodoptera frugiperda*, RNA sequencing, metabolism, plant defense

## Abstract

Sugarcane is the most important sugar crop, contributing ≥80% to total sugar production around the world. *Spodoptera frugiperda* is one of the main pests of sugarcane, potentially causing severe yield and sugar loss. The identification of key defense factors against *S. frugiperda* herbivory can provide targets for improving sugarcane resistance to insect pests by molecular breeding. In this work, we used one of the main sugarcane pests, *S. frugiperda*, as the tested insect to attack sugarcane. Integrated transcriptome and metabolomic analyses were performed to explore the changes in gene expression and metabolic processes that occurred in sugarcane leaf after continuous herbivory by *S. frugiperda* larvae for 72 h. The transcriptome analysis demonstrated that sugarcane pest herbivory enhanced several herbivory-induced responses, including carbohydrate metabolism, secondary metabolites and amino acid metabolism, plant hormone signaling transduction, pathogen responses, and transcription factors. Further metabolome analysis verified the inducement of specific metabolites of amino acids and secondary metabolites by insect herbivory. Finally, association analysis of the transcriptome and metabolome by the Pearson correlation coefficient method brought into focus the target defense genes against insect herbivory in sugarcane. These genes include *amidase* and *lipoxygenase* in amino acid metabolism, *peroxidase* in phenylpropanoid biosynthesis, and *pathogenesis-related protein 1* in plant hormone signal transduction. A putative regulatory model was proposed to illustrate the sugarcane defense mechanism against insect attack. This work will accelerate the dissection of the mechanism underlying insect herbivory in sugarcane and provide targets for improving sugarcane variety resistance to insect herbivory by molecular breeding.

## 1. Introduction

Sugarcane is the most important sugar crop, providing about 80% of sugar production worldwide [1]. During the growth stage of sugarcane, insect pests are a huge threat to sugarcane production. Insect herbivory often elicits a phytohormone-based defense response. Generally, chewing herbivory-induced responses are mediated by jasmonic acid (JA), while piercing/sucking insects elicit salicylic acid (SA)-mediated plant defenses [2,3]. JA, along with its derivatives, is a conserved defense phytohormone in plants in response to herbivory [4]. The elicited JA signaling can activate the expression of many genes, further resulting in the biosynthesis of diverse bioactive compounds [5,6] such as alkaloids, protease inhibitors (PIs), and polyphenol oxidases (PPOs) [7,8], which are toxic, repellent, or antinutritional to insects [9,10,11,12]. Other signaling hormones, including abscisic acid (ABA) and ethylene (ET), also participate in herbivory-induced responses [13,14,15].

In addition, the concentration of many amino acids increases after herbivory, including several amines such as tyramine, putrescine, and octopamine, and shikimate-derived amino acids including tyrosine, phenylalanine, and tryptophan. These amino acid are precursors or intermediates not only for many defensive secondary metabolites, such as phenolics, alkaloids, and phenylpropanoid, but also for phytohormones such as auxin and JA, as well as indole, which can be responsible for antiherbivory defense [16,17,18,19,20]. Some defense-related pathways are enriched in plants after insect infestation, including phenylpropanoid biosynthesis, flavonoid biosynthesis, plant hormone signal transduction, and plant–pathogen interactions [21,22]. Cytochrome P450 superfamily genes, peroxidases, and heat shock proteins are both important factors for plants’ immediate response to insect damage [23,24,25,26,27,28]. Moreover, the genes encoded by *fatty acid reductase* (*FAR*) and *heat shock protein 90KDA* (*Hsp90*) are also induced by insect herbivory [23,26,27,28,29]. After suffering from noctuid larvae attack, stronger protease inhibitor (PI) activity and a higher content of phenylpropanoid-derived metabolites were observed in plants [30,31]. In addition to this, insect herbivory can also activate the emission of volatile organic compounds, such as terpenoids and green-leaf volatiles, in response to this attack [11,32].

*Spodoptera frugiperda* is one of the main insect pests causing severe production loss to sugarcane; it mainly causes damages to the sugarcane leaf and thus leads to leaf blade incision [33,34]. Currently, *S. frugiperda,* as well as other sugarcane insect pests, are mainly controlled by chemical insecticides, which potentially cause environmental pollution [35]. The breeding of a new variety resistant to *S. frugiperda* is an efficient and ultimate way to reduce the damage caused by insect pests. Due to the time-consuming nature and low efficiency of conventional breeding programs, molecular breeding is a feasible way to breed a new variety with insect resistance, with great achievements obtained in trait improvement in other crops [36,37]. The elucidation of the mechanisms of and identification of the key defense genes against insects such as *S. frugiperda* can provide evidence and targets for improving sugarcane varieties with resistance to insects by molecular breeding.

Transcriptome analysis can identify a batch of genes related to a certain trait, and it has been successfully used in sugarcane to identify key genes associated with sucrose accumulation [38,39,40], disease response [41,42,43,44], and abiotic stress [45]. Meanwhile, the metabolome can provide dynamic changes in all metabolites in the organism, and it can interact with other omics, including the genome, transcriptome, and proteome, to modulate the biological process [46]. Thus, metabolomics associated with transcriptome analysis will help to more accurately identify the key genes related to a certain biological process.

In this work, to identify the defense genes against insect herbivory, we used one of the main sugarcane pests, *S. frugiperda*, as the tested insect to attack sugarcane. Then, the transcriptome was analyzed to identify the gene response to *S. frugiperda.* Meanwhile, metabolome analysis was also conducted to explore the metabolites triggered by this insect. Finally, association analysis was performed on the transcriptome and metabolome to identify the key genes that may confer resistance to *S. frugiperda* and other insect pests. This work will accelerate the dissection of the defense mechanisms underlying insect attack in sugarcane and provide targets for improving sugarcane variety with resistance to insect herbivory by molecular breeding.

## 2. Results

### 2.1. Overview of the Transcriptomic Analysis

**Figure 1:**
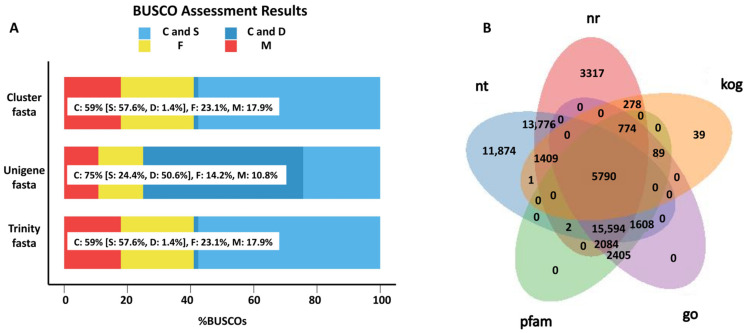
Overview of the transcriptomic analysis. (**A**) BUSCO analysis of the assembled transcripts. Light blue represents complete (C) and single copy (S); deep blue represents complete (C) and duplicated (D); yellow represents fragment (F); red represents missing (M). Trinity.fasta is the sequence assembled by the software Trinity. Cluster.fasta and unigene.fasta were obtained from trinity.fasta by removing the redundant sequence. (**B**) A Venn diagram of the genes annotated in different databases.

To uncover the gene responses to *S. frugiperda* herbivory in sugarcane, the transcriptome analysis of both treated and control leaves was conducted, and a total of ~133 million raw reads were generated. After filtering the adapter and low-quality reads, 38.27 Gb of clean data were obtained, with a Q30 of about 94%, and GC content ranging from 45.37% to 49.72% (Table S1). Based on these RNA sequences, 74,965 unigenes were assembled with an N50 of 1275 bp. The BUSCO analysis confirmed the high quality of the assembled transcripts (Figure 1A). Additionally, the BUSCO dataset was obtained from the website https://busco.ezlab.org/v2/datasets/embryophyta_odb9.tar.gz (accessed on 1 October 2022). By mapping these unigenes to the NR, NT, pfam, GO, and KOG databases, a total of 59,307 unigenes were annotated in at least one of these databases, and 5790 unigenes were annotated in all of these databases. In total, 43,024 unigenes were annotated in the NR database, 50,054 were annotated in NT, 23,696 were annotated in GO, 27,623 were annotated in pfam, 8380 were annotated in KOG, and 5790 genes overlapped in all five databases (Figure 1B). Moreover, 1608 unigenes were annotated as transcription factors.

### 2.2. Identification of Genes Related to Sugarcane Defense against S. frugiperda Herbivory by Transcriptome Analysis

**Figure 2:**
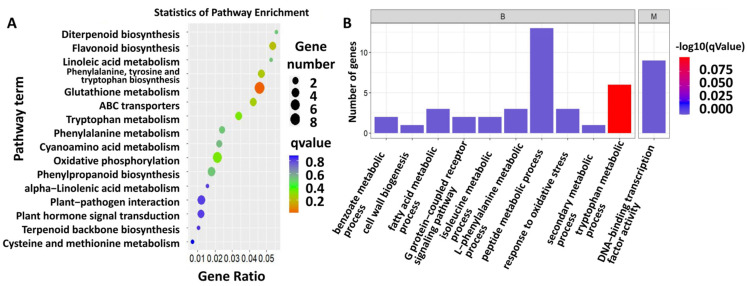
Function enrichment analysis of DEGs. (**A**) KEGG pathway enrichment of DEGs. Color indicates the degree of enrichment. Red represents stronger enrichment, green represents strong enrichment, blue represents enrichment. Gene ratio means the ratio of differential abundant proteins in this pathway accounting for total enriched proteins. (**B**) GO terms of DEGs.

A total of 482 differentially expressed genes (DEGs) were identified from sugarcane fed on by *S. frugiperda* (Table S2). Further KEGG analysis showed that the DEGs were enriched in the pathways related to flavonoid biosynthesis, linoleic acid metabolism, phenylalanine, tyrosine and tryptophan biosynthesis, glutathione metabolism, cyanoamino acid metabolism, oxidative phosphorylation, phenylpropanoid biosynthesis, plant–pathogen interaction, and plant hormone signal transduction (Figure 2A, Table S3). To obtain more information on the gene response to insect herbivory, GO analysis was also conducted on the DEGs, and the GO terms included peptide metabolic process, DNA-binding transcription factor activity, tryptophan metabolic process, L-phenylalanine metabolic process, response to oxidative stress, fatty acid metabolic process, G-protein-coupled receptor signaling pathway, isoleucine metabolic process, benzoate metabolic process, cell wall biogenesis, and secondary metabolic process (Figure 2B; Table S4).

To accurately identify the genes involved in sugarcane defense against insect herbivory, we analyzed the DEGs participating in the pathways related to plant defense response.

#### 2.2.1. Genes Associated with Biotic Stress Response

Genes related to biotic stress response were previously identified in plants infested by pests [47]. These genes may be associated with sugarcane defense against insect herbivory. We analyzed the expression pattern of these genes related to biotic stress in sugarcane between the treatment and control. Genes related to oxidoreductase activity and the fatty acid metabolic process are involved in plant response to biotic stress [48,49]. In the GO term “oxidoreductase activity”, the expression levels of most *cytochrome P450* and all *FAD-dependent oxidoreductase* were found to be induced by insect herbivory. A *long-chain-alcohol oxidase FAO2* and a *3-ketoacyl-CoA synthase* in the KEGG pathway “fatty acid metabolic process” were up-regulated in sugarcane fed on by insects. Moreover, insect attack up-regulated the expression of one *PTI1-like tyrosine-protein kinase* gene, one *WRKY* gene, and one *pathogenesis-related protein* gene involved in the KEGG pathway of plant–pathogen interaction. As proteinase inhibitor and heat shock proteins also play an important role in plant defense against insect herbivores [30,31,50], we also focused on the expression level of these genes, and we found that the expressions of two *heat shock 70 kDa protein* (*Hsp70*) genes were induced by insect herbivory, while three *cysteine proteinase inhibitor* genes were significantly suppressed in sugarcane after insect attack. In addition, eight *glutathione S-transferase* genes, which belonged to the oxidative stress response, were up-regulated in sugarcane fed on by insects (Table S5).

#### 2.2.2. Genes Involved in Secondary Metabolite Metabolism, Amino Acids, and Hormone Biosynthesis

Pathways related to secondary metabolite metabolism, amino acids, and hormone biosynthesis were also previously found to be responsible for pest attack response [16,17,18,19,20,21,22]. Thus, we also analyzed the genes involved in these pathways that were enriched by KEGG analysis.

In the secondary metabolite metabolism pathway of flavonoid biosynthesis, two *shikimate O-hydroxycinnamoyltransferase* genes and one *flavonoid 3’-monooxygenase* gene were induced by insect herbivory, while two *peroxidase* genes belonging to phenylpropanoid biosynthesis and one *all-trans-nonaprenyl-diphosphate synthase* gene in terpenoid backbone biosynthesis were down-regulated (Table S6).

In pathways related to amino acid biosynthesis or metabolism, insect herbivory induced one *amidase* and one *4-hydroxyphenylpyruvate dioxygenase* gene involved in phenylalanine metabolism; one *sarcosine oxidase*/*l-pipecolate oxidase* and one *hydroxypyruvate reductase* gene in glycine, serine, and threonine metabolism; one *arogenate/prephenate dehydratase* gene in phenylalanine, tyrosine, and tryptophan biosynthesis; and one *4-hydroxyphenylpyruvate dioxygenase* gene in tyrosine metabolism. In addition, we observed the down-regulation of two *3-deoxy-7-phosphoheptulonate synthase* genes involved in phenylalanine, tyrosine, and tryptophan biosynthesis; two *hydroxymandelonitrile lyase* genes in cyanoamino acid metabolism; one *acylpyruvate hydrolase* gene in tyrosine metabolism; and one *methylthioribulose 1-phosphate dehydratase/enolase-phosphatase E1* gene in cysteine and methionine metabolism (Table S6). These genes catalyze the biosynthesis or metabolism of amino acids, which are precursors and intermediates for secondary metabolites. Thus, these genes are directly or indirectly responsible for herbivory response.

With regard to hormone biosynthesis, we found that all these genes were induced, including one *gibberellin 2-beta-dioxygenase* gene involved in diterpenoid biosynthesis, one *cytokinin dehydrogenase* gene in zeatin biosynthesis, and one *lipoxygenase* in linoleic acid metabolism. However, one *auxin-responsive protein IAA* and one *transcription factor TGA* gene involved in plant hormone signal transduction were down-regulated (Table S6).

#### 2.2.3. Transcription Factors

Transcription factors (TFs) play an important role in plant response to abiotic and biotic stress [51,52,53]. In this work, we found that 25 transcription factors were differentially expressed in sugarcane after insect herbivory, with 19 up-regulated and 6 down-regulated. The up-regulated TFs comprised one *C2C2-Dof*, two *DBB*, one *TCP*, four *WRKY*, one *AP2/ERF-ERF*, four *NAC*, one *mTERF*, one *HB-BELL*, one *C2C2-CO-like*, one *MYB-related*, one *PLATZ,* and one other TF. The down-regulated TFs comprised one *AUX/IAA*, two *bZIP*, two *C2C2-Dof*, and one *NF-YC* (Table S7).

### 2.3. Validation of Candidate DEGs by qRT-PCR Analysis

**Figure 3:**
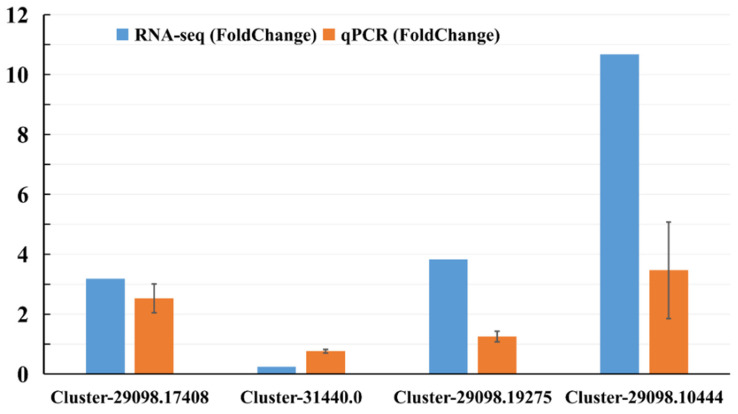
Gene expression levels from qPCR and RNA-seq. Orange columns represent the qPCR results, and blue columns represent the RNA-seq results. The *y*-axis represents the fold change in the relative expression level of the gene between the treated sample and control (HS vs. CS).

Quantitative reverse transcription–polymerase chain reaction analysis (qRT-PCR) was performed on four genes selected from the sequence database to validate the results of the Illumina RNA-seq. These genes were selected because they were most likely associated with defense response, and up- and down-regulated genes were included. The relative mRNA expression of unigenes assessed by qRT-PCR (Figure 3; Table S8) was very similar to the levels shown in the RNA-seq analysis, suggesting the reproducibility and accuracy of the RNA-seq results.

### 2.4. Overview of the Metabolomic Analysis

**Figure 4:**
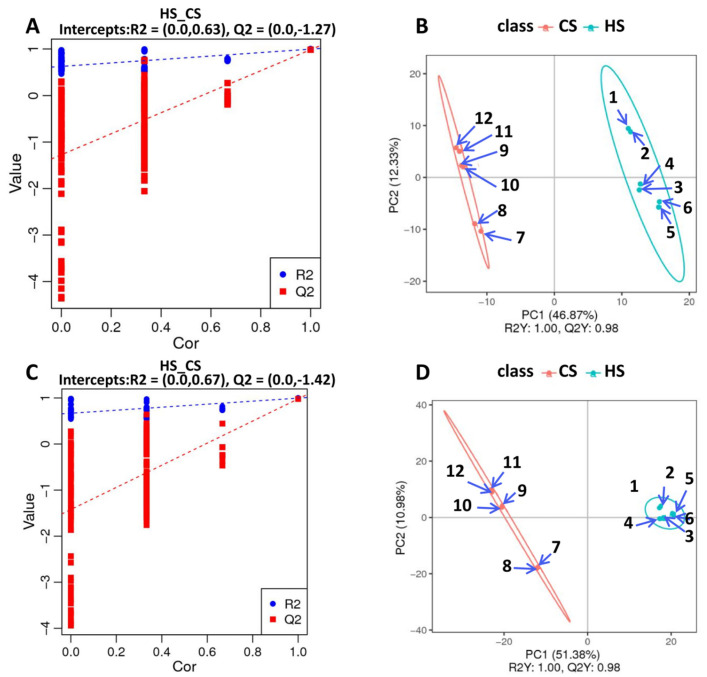
PLS-DA dispersion point diagrams and sorting verification diagrams: (**A**) PLS-DA dispersion point diagram (negative ion mode); (**B**) PLS-DA sorting verification diagram (negative ion mode); (**C**) PLS-DA dispersion point diagram (positive ion mode); (**D**) PLS-DA sorting verification diagram (positive ion mode).

**Figure 5:**
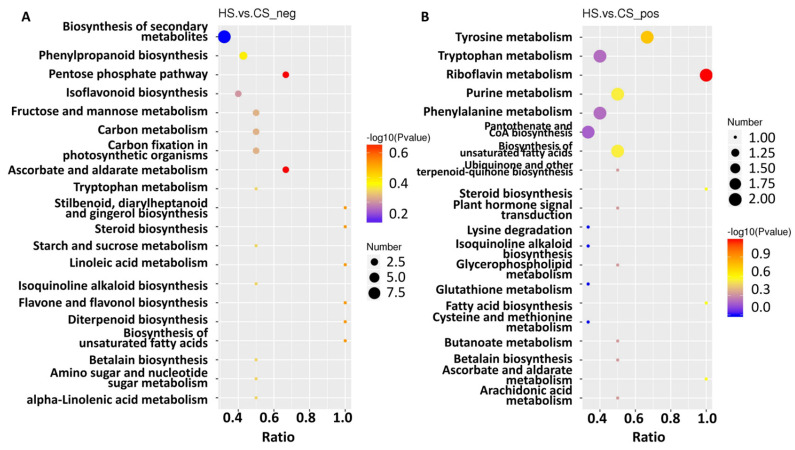
KEGG enrichment of the differential metabolites. (**A**) Metabolites identified in negative mode. (**B**) Metabolites identified in positive mode. The size of the dots corresponds to the number of DEGs in each pathway. The color displays the significance of enrichment. Ratio means the number of metabolites in this pathway with regard to the total enriched metabolite number.

To further explore metabolite changes in sugarcane triggered by *S. frugiperda* herbivory, metabolomic profiles were generated for the control and infested sugarcane. A total of 406 negative metabolites and 756 positive metabolites were detected from all samples (Table S9). Then, orthogonal partial least squares discriminant analysis (PLS-DA) showed good reproducibility of the different treatments (Figure 4A,B) and enabled further differential metabolite analysis. There were 155 differential metabolites in the negative group, with 76 up- and 79 down-regulated, and 279 differential metabolites in the positive group, with 139 up- and 140 down-regulated (Figure 4C,D; Table S9). KEGG analysis of these differential metabolites showed that they participate in the pathways related to linoleic acid metabolism, flavone and flavonol biosynthesis, phenylpropanoid biosynthesis, alpha-linolenic acid metabolism, isoquinoline alkaloid biosynthesis, isoflavonoid biosynthesis, and the biosynthesis of secondary metabolites (Figure 5A,B). These results indicate that amino acids and secondary metabolite metabolism are enriched in sugarcane under *S. frugiperda* stress.

### 2.5. Transcriptome and Metabolome Association Analysis to Identify Genes and Metabolites Related to Sugarcane Defense against S. frugiperda Herbivory

To identify the genes and metabolites with statistically significant correlation, the transcriptome and metabolome were integratively analyzed using the Pearson correlation coefficient method. The top 50 differential metabolites and the top 100 DEGs with significant correlations were identified (Tables S10 and S11) and subjected to KEGG analysis. The common pathways participated in by both differential metabolites and DEGs include glutathione metabolism, tyrosine metabolism, tryptophan metabolism, lysine degradation, cysteine and methionine metabolism, phenylalanine metabolism, alpha-linolenic acid metabolism, diterpenoid biosynthesis, linoleic acid metabolism, plant hormone signal transduction, phenylpropanoid biosynthesis, and ubiquinone and other terpenoid–quinone biosynthesis.

To further accurately identify the sugarcane genes and metabolites involved in the defense against *S. frugiperda*, and to elucidate the underlying mechanism, we analyzed the expression levels of these genes and their correlated metabolites in the pathways related to plant defense.

Amino acid metabolism has been reported to be involved in plant defense [16,17,18,19,20]. In the glutathione metabolism, tyrosine metabolism, and tryptophan metabolism pathways, the metabolites vitamin C, indole-3-acetamide, serotonin, rosmarinic acid, and levodopa were induced by herbivory. Correspondingly, eight *glutathione S-transferase* genes and one *4-hydroxyphenylpyruvate dioxygenase* gene were also induced and positively correlated with vitamin C and levodopa, respectively. Moreover, genes encoded by *catalase, amidase*, and *cytochrome P450 family 1 subfamily A polypeptide 1* were all induced and positively correlated with indole-3-acetamide and serotonin, while the *acylpyruvate hydrolase* gene was down-regulated and negatively correlated with rosmarinic acid. In the lysine degradation and cysteine and methionine metabolism pathways, genes encoding *sarcosine oxidase/l-pipecolate oxidase* and *methylthioribulose 1-phosphate dehydratase/enolase-phosphatase E1* were negatively correlated with metabolites N6,N6,N6-Trimethyl-L-lysine and L-cystine, respectively (Table 1). In phenylalanine, alpha-linolenic acid, diterpenoid, and linolenic acid metabolism, the *amidase*, *lipoxygenase*, *gibberellin 2-oxidase*, and *lipoxygenase* genes were positively correlated with benzoylformic acid, methyl jasmonate, gibberellin A7, and linoleic acid, respectively. Moreover, in the plant hormone signal transduction pathway, *auxin-responsive protein IAA* and *pathogenesis-related protein 1* were both negatively correlated with salicylic acid (Table 1).

In ubiquinone and other terpenoid–quinone biosyntheses, genes encoded by *4-hydroxyphenylpyruvate dioxygenase* and *apyrase* were negatively correlated with metabolites shikonin and hypoxanthine, respectively. In phenylpropanoid biosynthesis, *shikimate O-hydroxycinnamoyltransferase* was positively correlated with isoeugenol, *HCT shikimate O-hydroxycinnamoyltransferase* was negatively correlated with sinapoyl malate, and two genes encoding *peroxidase* were correlated with coniferin: one positively correlated, the other negatively correlated (Table 1). These genes and metabolites may also be involved in sugarcane defense against *S. frugiperda* herbivory.

## 3. Discussion

Sugarcane is a key sugar crop and one of the main energy crops globally. Fall armyworm (*Spodoptera frugiperda*) is a major sugarcane insect pest that can cause severe loss in sugarcane yield. The breeding of varieties resistant to insects such as *S. frugiperda* is an effective way to reduce the loss from insect damage. The elucidation of the mechanism underlying sugarcane defense against insect herbivory and the identification of the key defense genes is vital work to breed varieties with defense against insect herbivory by molecular breeding.

Transcriptome analysis is an efficient method to screen the genes related to a certain trait from an organism on a large scale, while metabolites are direct agents of plant defense against insect feeding and respond directly to pest attack. Metabolites are also direct or indirect products of gene expression [54,55,56]. Metabolome analysis can thus assist transcriptome analysis to more accurately identify the key genes related to a trait. Therefore, in this study, transcriptome and metabolome analyses were conducted simultaneously on sugarcane infested by a key insect pest, *S. frugiperda*. By association analysis of the transcriptome and metabolome, we identified 18 DEGs and 12 DMs (differential metabolites) from the pathway related to amino acid metabolism, including glutathione, tryptophan and tyrosine, cysteine and methionine, phenylalanine, alpha-linolenic acid, diterpenoid, and linoleic acid metabolism. In addition, one DM and three DEGs involved in plant hormone signal transduction were detected, as were three DMs and four DEGs attributed to phenylpropanoid biosynthesis.

Insect herbivory often elicits a phytohormone-based defense response. The up-regulation of defense-related pathways, including plant hormone signal transduction, was observed in switchgrass in response to fall armyworm [21]. JA and ET accumulation were observed in plants after infestation by a pest insect [57,58,59,60]. In this study, one *lipoxygenase* gene involved in linoleic acid metabolism was induced by pest attack. Increased *LOX* (*lipoxygenase*) activity levels have been reported in the interaction between plants and herbivores [61,62]. *LOXs* are also involved in the activation of downstream pathways including linoleic and linolenic acid and in regulating the induction of jasmonic acid (JA) [5,63,64]. In our work, one *methylthioribulose 1-phosphate dehydratase/enolase-phosphatase E1* gene involved in cysteine and methionine metabolism was down-regulated, indicating that expression changes in genes involved in ET and JA biosynthesis might be necessary for sugarcane defense against insect attack. These responses may be important for the acquisition of resources necessary for defense against insect herbivores [65,66]. Many DEGs in the JA signaling pathway, which regulates direct and indirect plant responses against herbivores [67,68,69], were also induced in our result.

The simultaneous induction of different hormonal signals results in cross-talk, which eventually leads to a specific defense response [13]. Genes involved in phenylpropanoid and flavonoid biosynthesis were induced in switchgrass in response to fall armyworm [21]. Furthermore, the activation of genes involved in flavonoid metabolism and plant–pathogen interactions may be associated with the resistance of cucumber to *Aphis gossypii* Glover (Hemiptera: Aphididae) [22]. These genes included transcripts coding for *phenylalanine ammonia-lyase* (*PAL*), *peroxidases*, *shikimate O-hydroxycinnamoyl transferases*, and *cinnamoyl-CoA reductase*, which are known to play a role in biotic stress defense responses [70,71,72]. In our work, many DEGs associated with secondary metabolism were also identified. Some genes in the flavonoid biosynthesis pathway were induced by herbivory, including two *shikimate O-hydroxycinnamoyltransferase* genes and one *flavonoid 3’-monooxygenase* gene. However, several genes belonging to phenylpropanoid biosynthesis or terpenoid backbone biosynthesis were down-regulated, such as two *peroxidase* genes and one *all-trans-nonaprenyl-diphosphate synthase* genes. Peroxidases are an important component of the immediate response of plants to insect damage [24,25]. The higher expression of *peroxidases* was reported previously, such as in grape in response to nonadapted strains of two-spotted spider mites [73] and in pigeon pea in response to cotton bollworm [61]. Moreover, analysis of the plant–pathogen interaction pathway showed that pest attack up-regulated one PTI1-like tyrosine-protein kinase, one WRKY, and one pathogenesis-related protein. These genes may constitute a part of the immunity system of sugarcane after pest infestation.

A number of amino acids increased after herbivory, including several amines (tyramine, putrescine, and octopamine) and shikimate-derived amino acids (tyrosine, phenylalanine, and tryptophan) [16,17,18,19,20]. In this study, many amino acid metabolism-related genes were found to be differentially expressed, including genes involved in phenylalanine, glycine, serine, threonine, tyrosine, tryptophan, and cyanoamino acid metabolism. These amino acids are precursors or intermediates of a large variety of defensive secondary metabolites, and some of them are also for phytohormones such as auxin and JA, as well as indole, which can prompt antiherbivore defense [16,17,18,19,20]. Thus, these identified genes might be necessary for producing antiherbivore defense metabolites.

Cytochrome P450 superfamily genes are another family of genes with oxidoreductase activity that are induced in response to WCM (wheat curl mite) herbivory [23,29,48]. The up-regulation of *fatty acid reductase (FAR)*-coding genes was detected in resistant wheat in response to WCMs [48]. It has also been reported that FAR1 improves resistance to oxidative stress and suppresses plant cell death [29,48]. In this study, in the GO term of oxidoreductase activity, the expression levels of most *cytochrome P450* and all *FAD-dependent oxidoreductase* genes were found to be induced in response to insect herbivory. In addition, eight *glutathione S-transferase* genes, which belonged to the oxidative-stress-related genes, as well as one *long-chain-alcohol oxidase FAO2* gene and one *3-ketoacyl-CoA synthase* gene involved in fatty acid metabolic process, were up-regulated in infested sugarcane. This result suggests that genes involved in oxidoreductase activity, oxidative stress response, and fatty acid metabolic process might be part of the response to insect herbivory.

Furthermore, four *heat shock protein 90*KDA (*Hsp90*)-coding genes were up-regulated in resistant wheat in response to WCM herbivory [29,48]. Heat shock proteins play a role in stress signal transduction [26,27,28]. The knockdown of *Hsp90* weakens the resistance of tomato to root-knot nematode and potato aphid [50]. Similarly, the expression of two *heat shock protein 70* KDA(*Hsp70*) genes was induced in sugarcane after fall armyworm damage, indicating that this *Hsp* is involved in sugarcane resistance to insect herbivory. After a noctuid larvae attack, stronger protease inhibitor (PI) activity and a higher content of phenylpropanoid-derived metabolites were found in plants [30]. In tomato, the enhancement of PI gene expression was found after moth oviposition [31]. As PI activity usually plays an important role in the defense against insect herbivores, we also focused on the expression level of proteinase inhibitor. However, three *cysteine proteinases inhibitors* were significantly suppressed in sugarcane after fall armyworm attack.

Transcription factors (TFs) play important roles in plant response to abiotic and biotic stress [51,52,53]. The *CmMYB19* gene in plants was found to be induced by aphid infestation [74]. *WRKY7*, *WRKY58*, *WRKY62*, *WRKY64*, and *WRKY76* were found to be expressed highly under rice blast disease [75]. The overexpression of rice *WRKY67* enhanced the resistance of transgenic rice plants to diseases [76]. The induction of NAC TFs was observed in response to greenbug infestation in sorghum [51]. bZIP proteins contribute to the defense response against Asian soybean rust disease (ASR) in soybean [77]. In our study, four *WRKY* TFs, four *NAC* TFs, and one MYB-related transcription factor were found to be induced by pest attack. Two *bZIP* TFs were suppressed. Thus, transcription factors *WRKY*, *NAC, MYB*, and *bZIP* might be involved in pest attack responses in sugarcane.

## 4. Materials and Methods

### 4.1. Plant Growth and Plant Infection

Sugarcane (*Saccharum* spp. interspecific hybrids) microshoots of variety GT44 were grown at the Sugarcane Research Institute (SRI), Guangxi Academy of Agricultural Sciences (GXAAS), Nanning, Guangxi, China. The field management was consistent with that described in our previous work [78]. Sugarcane was used in this study at the age of 2 months. *Spodoptera frugiperda* was also reared at SRI, GXAAS. Third-instar larvae were applied to the young sugarcane leaves using a brush. Sugarcane leaves without *S frugiperda* herbivory were used as controls. The infected and control sugarcane plants were placed in 2 nylon mesh cages, independently. Because the plant always respond within 72 h of attack [79], after 72 h, the insects were removed, and plants were used for RNA extraction or metabolite extraction. For transcriptome analysis, three biological replicates were set for both the control and infected treatments (CS1, CS2, CS3, HS1, HS2, HS3). CS represents a control sample, and HS denotes an infected sample. For metabolome analysis, six biological replicates were set for both treatments.

### 4.2. RNA Extraction, Library Construction, and Sequencing

RNA isolation was performed using a Plant RNA Kit (BioTeke, Beijing, China) according to the manufacturer’s instruction. RNA integrity was assessed using an Agilent 2100 bioanalyzer. The mRNA was enriched from total RNA using magnetic beads with Oligo (dT). The first cDNA strand was synthesized in the m-Mulv reverse transcriptase system using fragment mRNA as a template. Subsequently, the RNA strand was degraded by RNaseH, and the second cDNA strand was synthesized using dNTPs in the DNA Polymerase I system. Then, a Qubit2.0 Fluorometer was used for initial quantification, and the library was diluted to 1.5 ng/ul. An Agilent 2100 BioAnalyzer was then used to test the insert size of the library. Finally, paired-end reads were sequenced using an Illumina NovaSeq 6000 (Illumina Inc., San Diego, CA, USA).

### 4.3. Transcriptomic Data Analysis

Transcripts were assembled using the software Trinity [80]. Corset hierarchical clustering [81] was used to obtain the longest cluster sequence as unigenes for further analysis. The length of unigenes was statistically analyzed using customized Python scripts. BUSCO analysis were applied to assess the quality of assembled transcripts [82].

Unigenes were annotated using the software Blastx based on the NCBI nonredundant database and the Swiss-Prot, Clusters of Orthologous Groups (COG), and Kyoto Encyclopedia of Genes and Genomes (KEGG) databases. The cut-off was 10 × 10^−6^. The expression level was calculated by RSEM [83]. The fragments per kilobase of transcript per million (FPKM) value was used to present the expression level. DEGs were analyzed using DESeq2 [84] with a cut-off padj < 0.05 and fold change > 2. GOseq was used for GO enrichment analysis [85]. KOBAS was applied for KEGG enrichment analysis [86].

### 4.4. Quantitative Real-Time PCR Validation

The relative expression of mRNA was quantified via quantitative reverse transcription polymerase chain reaction (qRT-PCR) assay using sugarcane GAPDH (EF189713) mRNA as a reference, as designed by Ling [87]. qRT-PCR was performed using Bio-Rad SYBR Green PCR Master Mix (TaKaRa, Mountain View, CA, USA), with three biological replicates for each gene and three technical repeats per experiment. Target-specific primers were designed from RNA-seq sequences using the NCBI primer designer. The relative gene expression was calculated using the 2^−∆∆CT^ formula. The primers used in this study are listed in Supplementary Material Table S8.

### 4.5. Extraction and Quantification of Metabolites

Metabolites were extracted from leaves, with six biological replicates for both the infected and control treatments. Samples were ground using a grinder, and 100 mg of powder was extracted with 70% methanol at 4 °C for no less than 10 h (extraction liquid volume V = sample net weight (mg) × 12 μL/mg), then centrifuged at 10,000× *g* at 4 °C for 12 min. The supernatant was filtered by a microporous membrane (SCAA-104, 0.22 μm pore size, ANPEL, Shanghai, China). The filtered supernatant was used for further LC-MS system analysis [88].

### 4.6. Metabolomic Data Analysis

Firstly, the original file (.raw) obtained via mass spectrometry detection was imported into the Compound Discoverer 3.1 software (Thermo Fisher Scientific, MA, USA), and spectrogram processing and database searches were carried out to obtain qualitative and quantitative results for the metabolites; then, data quality control was carried out to ensure the accuracy and reliability of the data results. Multivariate statistical analysis of the metabolites, including principal component analysis (PCA) and partial least square discriminant analysis (PLS-DA), was conducted to reveal the differences in metabolic patterns among different groups. Hierarchical clustering (HCA) and correlation analysis of the metabolites were used to reveal the relationships between the samples and metabolites. Finally, the biological function of the metabolites was explained by the functional analysis of metabolic pathways. The main databases for the functional and classification annotation of the identified metabolites were KEGG, LIPID MAPS, etc. By using these databases, the identified metabolites were annotated in order to understand their functional characteristics and classification [89].

### 4.7. Metabolomic and Transcriptomic Association Analysis

The Pearson correlation coefficient was used to analyze the correlations between the differentially expressed genes from transcriptome analysis and the differential metabolites from metabolome analysis. The expression levels of genes from three biological samples of transcriptome combined with the quantitative value of triplicate biological samples from the metabolome were used for correlation analysis. If the correlation coefficient was less than 0, the correlation was negative, and vice versa. The top 50 differential metabolites and the top 100 DEGs, sorted from small to large by P value, were displayed. The differential genes and metabolites obtained were mapped to the KEGG pathway database synchronously to obtain their common pathway information.

## 5. Conclusions

**Figure 6:**
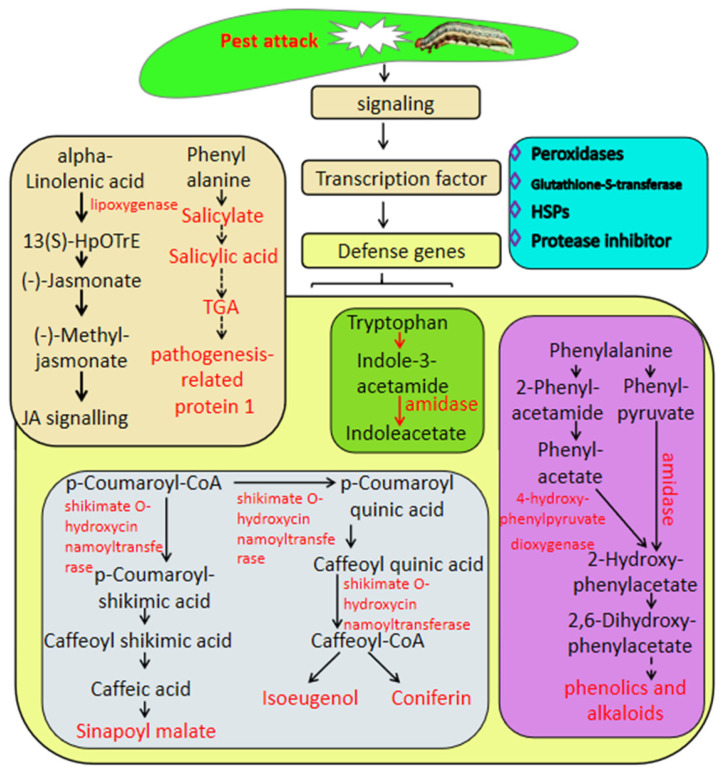
Putative model of sugarcane’s response to *Spodoptera frugiperda* herbivory. The names in light-type letters are metabolite compounds. The DEGs are exhibited in bold type with the arrows.

This work focused on the genes involved in the defense against insect herbivory in sugarcane by transcriptome analysis associated with metabolome analysis; the results showed that the defense response of sugarcane to pest is a complex process which constitutes expression changes in a high number of genes or metabolites involved in hormone biosynthesis and defense genes, including genes related to secondary metabolism, peroxidases, GSTs and heat shock proteins. The defense mechanism involves a number of TFs such as MYB, WRKY, and ERF, and signal transduction by various phytohormones such as salicylic acid and jasmonic acid (Figure 6). This work provides new insight into the mechanism underlying insect herbivory in sugarcane and provides candidate targets for the breeding of sugarcane varieties with resistance to insect herbivory by molecular technology.

## Figures and Tables

**Table 1:** Genes and their corresponding interacting metabolites involved in sugarcane defense against *S. frugiperda* herbivory.

Category	Pathways	Metabolites	Genes
Amino acid metabolism	Glutathione metabolism	Vitamin C (Com_3994_pos)	*glutathione S-transferase (Cluster-29098.25968|Cluster-29098.21080|Cluster-29098.11270|Cluster-29098.18423|Cluster-29098.4396|Cluster-29098.3024|Cluster-29098.25262|Cluster-29098.23558)*
	Tyrosine metabolism	Rosmarinic acid (Com_12324_pos), Levodopa (Com_3768_pos)	*4-hydroxyphenylpyruvate dioxygenase (Cluster-6018.1), acylpyruvate hydrolase (Cluster-29098.7692)*
	Tryptophan metabolism	3-hydroxyanthranilic acid (Com_1934_neg), Indole-3-acetamide (Com_11340_pos), Serotonin (Com_2896_pos)	*catalase (Cluster-29098.19669), amidase (Cluster-29098.16578), cytochrome P450 family 1 subfamily A polypeptide 1 (Cluster-29098.28485)*
	Lysine degradation	N6,N6,N6-Trimethyl-L-lysine (Com_1643_pos)	*sarcosine oxidase / L-pipecolate oxidase (Cluster-29098.10329)*
	Cysteine and methionine metabolism	L-cystine (Com_556_pos)	*methylthioribulose 1-phosphate dehydratase/enolase-phosphatase E1 (Cluster-29098.29499)*
	Phenylalanine metabolism	Benzoylformic Acid (Com_693_pos)	*amidase (Cluster-29098.16578)*
	Alpha-linolenic acid metabolism	Methyl jasmonate (Com_194_neg)	*lipoxygenase (Cluster-29098.17408)*
	Diterpenoid biosynthesis	Gibberellin A7 (Com_4755_neg)	*gibberellin 2-oxidase (Cluster-37110.0)*
	Linoleic acid metabolism	Linoleic acid (Com_1529_neg)	*lipoxygenase (Cluster-29098.17408)*
Plant hormone signal	Plant hormone signal transduction	Salicylic acid (Com_1139_pos)	*pathogenesis-related protein 1 (Cluster-31440.0)| auxin-responsive protein IAA ( Cluster-29098.10671)| transcription factor TGA (Cluster-29098.10444)*
Secondary metabolite biosynthesis	Phenylpropanoid biosynthesis	Isoeugenol (Com_1185_neg), Sinapoyl malate (Com_1638_neg), Coniferin (Com_4770_neg)	*shikimate O-hydroxycinnamoyltransferase (Cluster-29098.19275)| HCT shikimate O-hydroxycinnamoyltransferase ( Cluster-29098.4690)| peroxidase (Cluster-29098.6988, Cluster-29098.22224)*
	Ubiquinone and other terpenoid–quinone biosynthesis	Shikonin (Com_8894_pos)	*4-hydroxyphenylpyruvate dioxygenase (Cluster-29098.7692)*

## Data Availability

The raw RNA-Seq data of this study were deposited in the Genome Sequence Archive at the China National Center for Bioinformation (https://www.cncb.ac.cn/ (accessed on 10 October 2022)) under project number PRJCA009345.

## Supplementary Materials

The following supporting information can be downloaded at: https://www.mdpi.com/article/10.3390/ijms232213712/s1.
